# The type of endometrial preparation for embryo transfer after egg donation affects obstetric outcomes and the expression of placental angiogenic biomarkers

**DOI:** 10.1186/s12958-025-01521-w

**Published:** 2026-01-03

**Authors:** Andrea Roberto Carosso, Alessandro Rolfo, Valeria Maria Savasi, Enrico Papaleo, Laura Moretti, Anna Maria Nuzzo, Marco Carosso, Gianvito Contangelo, Ilaria Stura, Maria Elena Iacovazzi, Alberto Revelli, Gianluca Gennarelli

**Affiliations:** 1 Obstetrics and Gynecology 1U, Physiopathology of Reproduction and IVF Unit, Department of Surgical Sciences, Sant Anna Hospital, University of Turin, Turin, 10126 Italy; 2 Department of Surgical Sciences, Gynecology and Obstetrics 2, City of Health and Science-S. Anna University Hospital, University of Turin, Turin, 10126 Italy; 3 Department of Woman Mother and Neonate, Buzzi Children’s Hospital, University of Milan, Milan, 20124 Italy; 4 Gynecological Obstetric Department, San Raffaele Hospital, University of Milan, Milan, 20124 Italy; 5 Department of Neurosciences, University of Turin, Turin, 10126 Italy

**Keywords:** Endometrial preparation, Frozen embryo transfer (FET), Natural cycle, Obstetric outcome, Oocyte donation, Placenta

## Abstract

**Background:**

We investigated the impact of natural cycle/modified natural cycle and artificial cycle in oocyte donation pregnancies on obstetric/neonatal outcomes and placental angiogenic biomarkers.

**Methods:**

A total number of 201 singleton live births resulted from in vitro fertilization with oocyte donation were enrolled: *n* = 70 after natural cycle/modified natural cycle endometrial preparation and *n* = 131 after artificial cycle endometrial preparation. Moreover, 35 placental biopsies were collected: *n* = 12 after natural cycle/modified natural cycle endometrial preparation, *n* = 23 after artificial cycle endometrial preparation. Finally, 24 placentae from women with a spontaneous, healthy singleton pregnancy at term, who showed no signs of maternal, placental or fetal disease were used as control.

**Results:**

We reported a lower incidence of both hypertensive disorders of pregnancy (7.1% Vs 18.3%, *p* < 0.05), including preeclampsia, and placental previa (0 Vs 6.1%, *p* < 0.05) in natural/modified natural cycles compared to artificial cycle. Furthermore, better neonatal outcomes, at least in terms of low birth weight (5.7% Vs 20.9%, *p* < 0.05) and intensive care unit admission (2.9% Vs 15.6%, *p* < 0.05), were observed. From a molecular point of view, a significant gene over-expression of pro-angiogenic placental growth factor and vascular endothelial growth factor were obtained in natural cycles. Conversely, anti-angiogenic Soluble Fms-Like Tyrosine Kinase- 1 levels were increased in artificial cycle group placentae compared to natural/modified natural cycle and controls.

**Conclusions:**

Natural/modified cycles should be promoted as preferential approach for endometrial preparation in oocyte donation pregnancies, at least when regular (or inducible) ovulatory cycles are present.

## Background

In vitro fertilization (IVF) with oocyte donation (OD) has emerged as a groundbreaking solution for couples facing infertility issues, particularly for women in advanced reproductive age and/or with very poor ovarian reserve. Whereas OD represents an excellent procedure in terms of pregnancy rates, it is commonly acknowledged that OD pregnancies show increased obstetric/neonatal complications [1], such as hypertensive disorders of pregnancy (HDP) including pre-eclampsia (PE), post-partum haemorrage (PPH), preterm birth (PTB), small for gestational age (SGA) and low birth weight (LBW) newborns [2]. Several mechanisms are currently suggested as putative pathogenetic factors for such complications; a frequently candidate is immunological maladaptation, associated with the full allogenic nature of OD pregnancy [3], with consequent abnormal placentation and increased local inflammation.

A similar increase in the same complications has been reported by several studies even in autologous IVF pregnancies, specifically when obtained after frozen embryo-transfer (FET) [4, 5]. Accumulating evidence supports the notion that the increased risk observed in autologous pregnancies from FET is essentially related to the type of endometrial preparation, rather than to embryo cryopreservation per se [6].

Currently, strategies for endometrial preparation in case of FET are represented by either natural cycle (NC)/modified natural cycle (m-NCs) or artificial cycle (ACs). In AC, the endometrium is prepared via the administration of exogenous estrogens and progesterone, and ovulation does not occur [7]: the absence of a corpus luteum (CL) has been suggested as a relevant pathogenetic mechanism behind the increased incidence of obstetric complications. Indeed, substances normally produced by the CL are involved in the physiology of uterine/systemic adaptation to pregnancy [8]. At molecular level, the generalized endothelial dysfunction typical of HDP is directly related to the unbalanced production of placental pro- and anti-angiogenic molecules, including placental growth factor (PlGF) and soluble FMS-like tyrosine kinase-1 (sFlt-1) [9, 10]. During normal placental development, vascular endothelial growth factor (VEGF) and PlGF regulate villous angiogenesis and remodeling of maternal spiral arteries [9, 10]. Conversely, abnormal expression of sFlt-1 inhibits VEGF and PIGF biological functions, induce endothelial dysfunction and placentation anomalies [11, 12].

Whatever the mechanisms involved, the evidence for a higher incidence of HDP after AC in autologous IVF is robust [13–15]. Differently, data in OD cycles are lacking. Herein, we investigated the impact of NC/m-NC and AC in OD pregnancies on obstetric/neonatal outcomes. The expression of placental angiogenic biomarkers among different strategies of endometrial preparation was also evaluated, and potential correlations to clinical outcomes were explored.

## Methods

This was a prospective, observational, multicenter cohort study, in which clinical data and biological samples were collected at Sant’Anna Hospital (Turin, Italy), San Raffaele Hospital (Milan, Italy), and Sacco Hospital (Milan, Italy) between January 2022 and March 2024.

The study was performed in adherence to the principles expressed in the Declaration of Helsinki. Patient’s recruitment was performed in accordance with O.I.R.M. S. Anna Hospital and Ordine Mauriziano di Torino Ethical Committee (CS2/1218; Protocol. n. 0053289). All patients provided written informed consent.

Patients undergoing an IVF/intracytoplasmic sperm injection (ICSI) cycle with OD (either with fresh or frozen ET), resulting in a singleton live birth (> 22week gestation), were included in the study.

Early pregnancy losses (< 22 weeks of gestation), including biochemical pregnancies and miscarriages, were excluded from the analysis because placental tissue and comprehensive obstetric data were not available. However, all cases were recorded in the database and their exclusion was applied uniformly to both study groups to minimize selection bias.

Patients were assigned to either the NC/m-NC or AC protocol according to clinical indications and center-specific practices, rather than by randomization. In particular, NC/m-NC cycles were preferred in women with regular ovulatory cycles, whereas AC was adopted for patients with anovulation, premature ovarian failure, or logistical constraints preventing synchronized embryo transfer.

To ensure comparability, only singleton live births after OD-IVF performed in the same time period and under identical inclusion/exclusion criteria were analyzed. The two groups were subsequently compared for baseline characteristics (maternal age, BMI, gynecological comorbidities, and smoking habits), as summarized in Tables 1 and 2.

**Table 1:** Demographic characteristics in the study groups

	NC/m-NC (*n* = 70)		AC (*n* = 131)		*p*-value
	Mean	SD	Mean	SD	
Age (years)	43.9	3.4	43.7	3.5	0.6147
Weight (Kg)	59.7	9.1	60.1	9.3	0.6966
BMI	22.2	3.8	22.1	3.1	0.6093
Weight gain (Kg)	11.4	3.9	10.6	4.5	0.3627
Donor Age (years)	25.6	3.8	25.8	4.1	0.7455

**Table 2:** Clinical variables and treatment-related variables in the study groups. ASA = acetylsalicylic acid

	NC/m-NC (*n* = 70)		AC (*n* = 131)		
	*N*	%	*N*	%	*p* value
Cigarette Smoking	1	1.43	14	10.69	**0.0173**
Gynecological comorbidities (leiomyomas)	19	27.14	24	18.32	0.1462
Previous abortion	27	38.57	51	38.93	0.9602
Ethnicity					
Caucasian	68	97.14	122	93.85	0.4513
Asiatic	0	0	3	2.31	
African	1	1.43	1	0.77	
South American	0	0	3	2.31	
Other	1	1.43	1	0.77	
ASA during pregnancy	66	94.29	113	86.26	0.0825
Heparin during pregnancy	6	8.57	17	12.98	0.3499
Chronic hypertension	3	4.29	9	6.87	0.4613
Indication for oocyte donation					
Advanced maternal age	23	32.9	52	39.7	0.34
Reduced ovarian reserve	29	41.4	47	35.9	0.45
Repeated failures of homologous IVF	12	17.1	12	9.2	0.10
Endometriosis	2	2.9	11	8.4	0.15
Poliabortivity	4	5.7	9	6.9	0.75

Bold values indicate statistical significance (*p* < 0.05)

Moreover, cases with twin pregnancy, late abortion, or therapeutic termination of pregnancy were excluded from the statistical analysis.

Endometrial preparation was either NC/m-NC or AC, as described elsewhere [7].

The variables relating to the patients’ characteristics and treatment are resumed in Tables 1 and 2.

The following obstetric outcomes/pathologies were considered: gestational age at delivery, delivery mode (spontaneous, operative, or Cesarean section (CS), PPH, defined as blood loss > 500 mL after a vaginal delivery and > 1000 mL after CS, PE, pregnancy-induced hypertension (PIH), gestational diabetes (GDM), preterm premature rupture of membranes (pPROM), chorioamniotitis, days of maternal hospitalization, threatened preterm delivery, labour induction, arterial neonatal pH at birth, placental weight and anomalies (abnormal localization/insertion; placental retention after birth). Pregnancy-associated plasma protein (PAPP-A) levels in the first trimester (collected during the routine pre-natal diagnostic workout) were also evaluated.

The following fetal/neonatal outcomes were considered: early preterm birth (< 32 weeks), late preterm birth (32–37 weeks), low birth weight (< 2500 g), small for gestational age (SGA, < 10 centile considering gestational age and sex), large for gestational age (LGA, > 90 centile considering gestational age and sex), intrauterine growth restriction (IUGR), neonatal macrosomia (birthweight > 4000 g), APGAR score ≤ 7 at 5’, and admission to the Neonatal Intensive Care Unit (NICU).

### Placenta tissue collection

Only placentas from births taking place at the S. Anna hospital were collected in order to avoid potential bias related to cryopreservation and shipping of placental material.

The study included placentas from OD pregnancies after NC/mNC endometrial preparation (*n* = 12), or AC endometrial preparation (*n* = 23). The analysis included also a control group (CTRL; *n* = 24) composed of women with a spontaneous, healthy singleton pregnancy at term, who showed no signs of maternal, placental or fetal disease. Placental biopsies were randomly collected from the basal plate and snap frozen immediately after delivery. The biopsies were then processed for mRNA and protein isolation. Calcified, necrotic and damaged areas were excluded from collection.

### RNA isolation and real time PCR

Total RNA was isolated from frozen placental biopsies using TRI^®^ reagent (Sigma-Aldrich, Italy) according to the manufacturer’s instructions and then treated with DNAse I to remove genomic DNA contamination. Three µg of total RNA were reverse transcribed using a random hexamers approach (Fermentas, Germany) and a RevertAid H Minus First Strand cDNA synthesis kit (Fermentas, Germany). Gene expression levels of VEGF, PlGF and s-Flt1 were determined by real-time PCR (Applied Biosystems, USA) using specific TaqMan primers and probes following the manufacturer’s protocol (Life Technologies, USA, ). Ribosomal 18 S and GAPDH RNA expression was used as an internal reference (Life Technologies, USA) and relative expression and fold change were calculated according to Livak and Schmittgen.

### Protein isolation and Enzyme-Linked Immunosorbent (ELISA) assay

Total proteins were isolated from placental biopsies using 1X Radio Immunoprecipitation Assay (RIPA) buffer supplemented with Protease Inhibitors. PlGF, s-Flt1 and VEGF placental levels were determined using commercially available competitive ELISA kit (RayBiotech, USA) according to the manufacturer’s instruction. The absorbance was measured at 450 nm using an ELISA SR 400 microplate reader (Biorad Laboratories, Italy).

### Statistical analysis

An a priori sample size calculation was done using PROC POWER by SAS^®^ 9.4: 176 patients resulted as a sufficient number to see a difference of 10% in HDP with a power of 80%. Considering a posteriori sample size of 201 women, the final power of this study was 85%. A sample size calculation on the number of placental biopsies was not possible because the expected difference of SFLT1, PIGF and VEGF was unknown among the different groups. Therefore, the collection of placental biopsies was performed in a given period of time, from December 2022 to February 2024.

The results of descriptive statistics were expressed as absolute numbers (n) or percentages (%) for categorical variables, while continuous variables were reported as means and standard deviations (SDs). Gestational age was reported via the median and interquartile range (IQR). Either Student’s t-test or the Wilcoxon–Mann–Whitney test were used to compare continuous variables, according to the type of distribution. The Chi-squared test and Fischer’s exact test were used to compare categorical variables.

Odds Ratios (OR) were performed to deepen the differences between cycles. In particular, multivariate analysis were done to adjust the results by smoking, maternal age, BMI, and the use of heparin and aspirin during pregnancy. Statistical analysis was performed using the SPSS Statistics software, version 28.0.1.0, and SAS for Windows, version 9.4. A p value < 0.05 was considered statistically significant.

## Results

A total number of 201 singleton live births resulted from IVF with OD: *n* = 70 after NC/m-NC endometrial preparation and *n* = 131 after AC endometrial preparation. All centers used frozen donor oocytes for both fresh and frozen donor ETs. Most oocyte donors were in their 20s or early 30s, anonymous and predominantly Caucasian. All ETs were performed at the blastocyst stage.

Demographic characteristics, clinical variables and treatment-related variables in NC/m-NC and AC groups of pregnant women are reported in Tables 1 and 2. The groups were comparable with respect to maternal age, ethnicity, BMI, gynecological comorbidities, indication for oocyte donation whereas they differed in smoking habit before pregnancy. A subtle difference was registered in endometrial thickness at ET.

### Obstetric outcomes

Both the risks of HDP and abnormal placental insertion were significantly increased the in the AC group (Table 3). PIH was significantly more represented, and PE tended to be more frequent among women in the AC group, with a relevant difference in the absolute number of cases. These differences were matched by differences in the length of hospitalization and in the proportion of CS (Table 3). The two study groups did not differ in gestational age, incidence of GDM, pPROM and chorioamniotitis (Table 3).

**Table 3:** Comparison of obstetric outcomes between the study groups. Continuous variables are reported as mean and standard deviation (SD) if not otherwise indicated. Discrete variables are reported as n (%)

	NC/m-NC (*n* = 70)	AC (*n* = 131)	*p*-value
Gestational age (weeks). median (Q1:Q3)	39 (38:39)	38.3 (37:39)	0.0582
PAPP-A (mom)	1.36 (0.68)	1.12 (0.60)	0.0743
Placenta weight (gr)	547.2 (107.8)	544.06 (139.6)	0.606
Birthweight (gr)	3144.5 (433.5)	2994.1 (595.2)	0.1574
Neonatal arterial pH at birth	7.27 (0.06)	7.29 (0.08)	0.1469
Maternal hospitalization (days)	5.07 (4.4)	5.79 (3.03)	**0.0023**
Type of delivery			
Spont. vaginal delivery	29 (41.4)	29 (22.5)	**0.013**
Operative vaginal delivery	2 (2.9)	2 (1.6)	
C-Section	39 (55.7)	98 (76.0)	
Labour induction	23 (32.9)	29 (22.1)	0.0983
Preterm premature rupture of membranes (pprom)	6 (8.6)	6 (4.6)	0.2552
Chorioamniotitis	0 (0)	0 (0)	-
Postpartum haemorrage (PPH) (> 500 ml after vaginal delivery. >1000 ml after c-section)	10 (14.3)	23 (18.1)	0.4915
Placenta previa	0 (0)	8 (6.1)	**0.0349**
Threatened preterm labor	2 (2.9)	13 (1.0)	0.0674
Pregnancy-induced hypertension (PIH)	5 (7.1)	24 (18.3)	**0.0317**
Pre-eclampsia (PE)	1 (1.4)	10 (7.6)	0.0654
Gestational diabetes (GDM)	12 (17.1)	16 (12.2)	0.3363

Bold values indicate statistical significance (*p* < 0.05)

### Neonatal outcomes

The AC subgroup showed a higher incidence of cases with both low birth weight and NICU hospitalization (Table 4). All the other neonatal outcomes were comparable between the two groups.

**Table 4:** Comparison of neonatal outcomes between the study groups. Variables are reported as n (%)

	NC/m-NC (*n* = 70)	AC (*n* = 131)	*p*-value
Late preterm (32-37w)	5 (7.1)	20 (15.6)	0.0928
LBW (< 2500 g)	4 (5.7)	27 (20.9)	**0.0047**
Macrosomia ≥ 4000 gr	8 (11.4)	8 (6.1)	0.1842
SGA	0 (0)	2 (1.54)	0.297
IUGR	2 (2.9)	5 (3.8)	0.7237
APGAR ≤ 7 at 5’	70 (100)	123(93.9)	**0.0349**
Neonatal Intensive Care Unit admission	2 (2.9)	20 (15.6)	**0.0068**

Bold values indicate statistical significance (*p* < 0.05)

### Placental angiogenic biomarkers

**Fig. 1:**
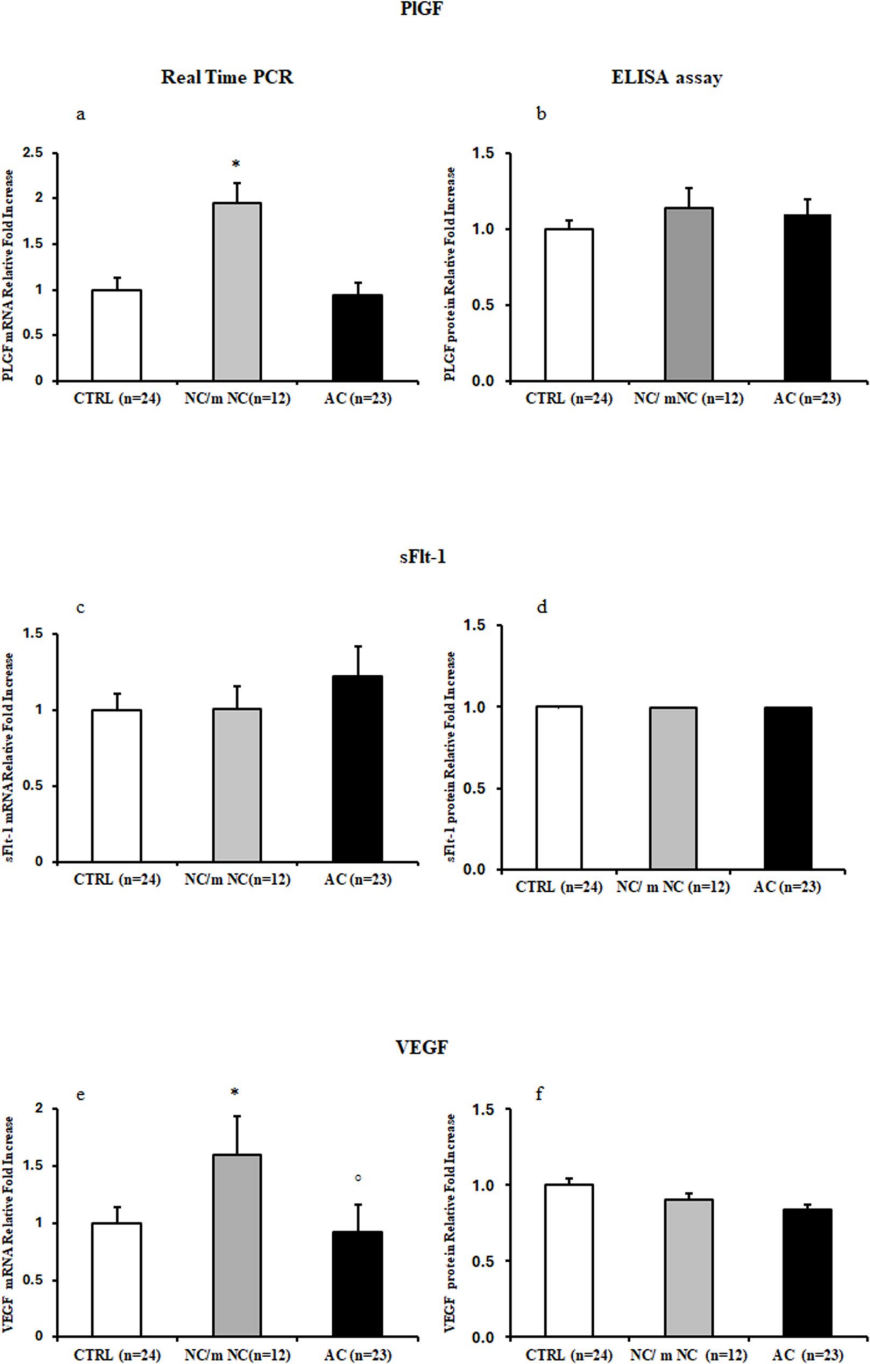
PlGF, sFlt-1 and VEGF gene and protein expression levels in CTRL, NC/m-NC and AC placentae. **(a)** mRNA and (**b**) protein expression of PlGF in CTRL, NC/m-NC and AC placentae; (**c**) mRNA and (**d**) protein expression of sFlt-1 in CTRL, NC/m-NC and AC placentae; (**e**) mRNA and (**f**) protein expression of VEGF in CTRL, NC/m-NC and AC placentae. Statistical significance has been considered as *p* < 0.05. *P < 0.05 versus CTRL and °*P* < 0.05 versus NC/m-NC

Pro-angiogenic PlGF and VEGF mRNA expression levels were significantly increased in NC/m-NC relative to CTRL placentae (*p* < 0.001, 1.9-Fold Increase; *p* = 0.013, 1.6-Fold Increase, respectively) while no significant differences were reported in AC relative to CTRL group (*p* > 0.05) (Figs. 1a and e). Furthermore, VEGF mRNA levels were significantly decreased in AC placentae relative to NC/m-NC group (*p* = 0.017, 0.6-Fold Decrease) (Fig. 1e). A trend toward increased anti-angiogenic sFlt1 gene expression levels were found in AC relative to CTRL placentae (1.2 Fold Increase), while no significant differences were reported in NC/m-NC relative to CTRL (*p* > 0.05) (Fig. 1c). At protein level, PlGF showed a trend of increase in NC/m-NC and AC relative to CTRL placentae (1.14 and 1.10-Fold Increase respectively) (Fig. 1b) while no differences were found in VEGF and sFlt1 protein expression among groups (*p* > 0.05) (Figs. 1d and f).

### Confounding factors: smoke

In our sample, 15 patients smoke (7.46%), with a significant difference in the two groups (1.43 NC, 10.69 AC, *p* = 0.0173, OR = 8.2 (1.0–64.1)). For this reason, we conducted a multivariate analysis (OR) considering the smoke as confounding factor. No changes were seen in the multivariate analysis, in particular considering the significant variables, as reported in Table 5.

**Table 5:** Simple and smoke-adjusted odds ratios (OR) for maternal and neonatal outcomes in natural cycle (NC) versus artificial cycle (AC) frozen embryo transfers. Odds ratios were calculated using univariate (simple) and multivariate (adjusted) logistic regression analyses. Adjustment was performed for smoking as a potential confounding factor. The adjustment did not modify the significance or direction of associations, confirming that smoking was not a confounder in this dataset

Outcome	Simple OR	95% CI	Adjusted OR (for smoke)	95% CI	Interpretation
Spontaneous vaginal delivery vs. C-section	0.398	0.211–0.751	0.373	0.195–0.715	Lower rate of vaginal delivery in AC group
Maternal hypertension	3.590	1.039–12.414	3.677	1.041–12.984	Higher risk of hypertension in AC group
Apgar score at 5’ >7 vs. ≤ 7	4.756	1.205–18.769	4.922	1.208–20.049	Higher rate of low Apgar in AC group
Low birth weight (< 2500 g)	4.366	1.461–13.045	4.518	1.504–13.577	Higher risk of LBW in AC group
NICU admission	6.204	1.405–27.393	6.224	1.401–27.653	Higher NICU admission rate in AC group

### Confounding factors: other variables

Other factors could be confounders in the analysis, in particular we controlled maternal age (OR = 0.98, 0.9-1.0), BMI (OR = 0.98, 0.9-1.0), use of aspirin during pregnancy (OR = 2.6, 0.85–8.09) and the use of heparin during pregnancy (OR = 1.6, 0.6–4.2). However, these variables were not significant in the analysis, i.e. they were not confounding factors in our sample.

## Discussion

We reported a lower incidence of both HDP, including PE, and placental anomalies in NC/m-NC compared to AC cycles resulting in lower obstetric complications and better neonatal outcomes, at least in terms of low birth weight and NICU admission. We demonstrated a significantly increased gene expression levels of pro-angiogenic PlGF and VEGF in NC/m-NC compared to AC oocyte donation. Instead, anti-angiogenic sFlt-1 mRNA levels were increased in AC group relative to NC/m-NC and controls demonstrating a placental pro-angiogenic profile in NC/m-NC, thus emphasizing the potential beneficial role of CL for IVF success with OD pregnancy.

Since the first successful use of OD in 1984, an increasing number of couples use donor oocytes to treat infertility. In Europe, OD accounts for about 8% of all IVF cycles, with pregnancy rates per fresh ET reaching an average of 50.5% [16]. OD represents a major challenge for modern maternal-fetal medicine, due to the higher incidence of obstetric complications compared to both naturally-conceived and homologous IVF pregnancies. In particular, OD has been independently associated with a higher rate of PIH and PE, probably as a consequence of altered placental development [17].

For a long time [18] and still today [19], OD immunological aspects have been considered the suspect culprit for abnormal placentation and increased clinical complications. Whereas several factors are involved in placenta formation, an early and pivotal role is played by CL secretory function.

Beyond the known actions of progesterone and estradiol on the uterus, other molecules produced by the CL have been shown to exert angiogenic activities, which are involved in optimal implantation/placentation and initiation of a physiologic pregnancy. As an example relaxin, whose serum levels are undetectable in pregnant women without a CL, has been shown to play a significant role in maternal cardiovascular and renal adaptations to pregnancy [20]. Currently, the artificial hormonal preparation of the endometrium with exogenous estrogens and progesterone, which implies the absence of CL, represents the most common strategy in OD cycles [21]. Understanding the relative role of different endometrial preparations in the pathogenesis of obstetric/neonatal complications often associated with OD pregnancy would be of major interest. To our knowledge, this is the first study specifically focusing on this topic, including both clinical and molecular observations.

The main finding of the present study was a higher incidence of obstetric/neonatal complications in the AC group. From a pathogenetic standpoint, one possible explanation for the observed aberrations in AC cycles comes from the analysis of serum and placental biomarkers of obstetric pathology. Indeed, a tendency toward a lower concentration of PAPP-A (p 0.07) was observed in AC cycles; PAPP-A is a metalloproteinase considered to be essential for fetal and placental development [22]. Previous studies confirm an association between decreased first-trimester concentration of PAPP-A and abnormal placentation/placental dysfunction, which results in the development of PE [23]. The correlation between the CL activity and PAPP-a circulating concentration has been known for some time [24]; indeed, the CL expresses PAPP-A, and an higher concentration is observed during the secretory phase of the menstrual cycle [25]. It is therefore expected that in women with AC, PAPP-a levels in the first trimester are lower than in spontaneous pregnancies. However, this might not be the sole mechanism behind the observed complications.

Higher estrogen in the first trimester, as in AC cycles, has been associated to worse obstetric outcomes, such as pre-eclampsia [26]. Estradiol, in particular, plays a crucial role in placentation and may be involved in spiral arteries remodelling as previous demonstrated in animal model [27].

Furthermore, it was previously reported that IVF pregnancies are generally characterized by an anti-angiogenic profile with significantly higher concentration of placental sFlt-1, accompanied by lower levels of PlGF [28]. Nevertheless, only few studies specifically investigated sFlt1/PlGF expression in IVF pregnancies with or without CL, and data are conflicting. Conrad et al. showed increased sFlt-1 levels in both absent/present CL IVF pregnancies groups compared to spontaneously conceived controls, with higher sFlt-1/PlGF ratio in absent vs. present CL pregnancies [29]. In contrast, no differences in sFlt-1 and PlGF concentration between absent/present CL groups were found by Woo and colleagues [30].

No specific work has so far addressed the issue of angiogenesis biomarkers in placentae from OD pregnancy. Herein, we reported significantly increased gene expression levels of pro-angiogenic PlGF and VEGF in natural cycles OD compared to artificial cycles OD, while anti-angiogenic sFlt-1 mRNA levels were increased in AC group relative to NC/m-NC and controls. Contrary to what shown for gene expression, which stands for a “programming” of placental activity, no differences were observed at the protein level among groups. This finding could have more than one explanation. As a matter of fact, the small number of observations might limit the detection power. We acknowledge that this relatively limited sample size (12 vs. 23 placentae) represents a constraint of the study, as it may reduce the statistical power and findings generalizability. Nonetheless, our molecular data provide valuable mechanistic insight that supports the clinical observations. However, since placentas were studied at term, nothing could be said about protein expression during the first trimester (when the role of proangiogenic factors is crucial).

Our data, for the first time to our knowledge, demonstrate a placental pro-angiogenic profile in OD pregnancies with CL, thus emphasizing its potential beneficial role for the success of IVF-OD pregnancy. Moreover, our gene expression data suggest the existence of CL-related placental epigenetic modifications during early pregnancy, when the CL is active and the placenta is developing, opening to new investigation perspectives.

Importantly, the indications for oocyte donation did not differ significantly between the NC/m-NC and AC groups, thus reducing the likelihood that differences in the underlying medical indications for OD could have biased our findings. As shown in Table 2, the distribution of indications for OD was comparable between groups: advanced maternal age (32.9% vs. 39.7%, *p* = 0.34), reduced ovarian reserve (41.4% vs. 35.9%, *p* = 0.45), repeated failures of homologous IVF (17.1% vs. 9.2%, *p* = 0.10), endometriosis (2.9% vs. 8.4%, *p* = 0.15), and recurrent pregnancy loss (5.7% vs. 6.9%, *p* = 0.75).Therefore, the observed clinical and molecular differences between NC/m-NC and AC pregnancies cannot be attributed to discrepancies in the underlying cause of infertility.

We included a limited number of observations with an uneven distribution between groups. Indeed, it is currently difficult to enlarge numerically NC group as most centers worldwide perform IVF-OD using FET after AC preparation, a logistically more convenient solution, indispensable in women with anovulatory cycles or in menopause. We implemented data with placental molecular analysis to strength our observations offering a pathogenetic rationale.

In light of the present results, as already reported in homologous IVF pregnancies, our findings are hypothesis-generating and suggest a potential association between NC and more favorable obstetric outcomes compared to AC in OD pregnancies. While these data support the possible preferential use of NC when feasible, confirmation by randomized controlled trials is required before clinical recommendations can be made.

Although some patients who approach to OD need to be treated with ACs because of their premature ovarian failure, this is not true for women with regular menstruation. Furthermore, even for women with chronic anovulation, ovulation induction with selective estrogen receptor modulators (clomiphene, letrozole) or low-dose gonadotropins can be successfully applied, in order to provide the CL presence. Whereas the choice of extensively apply NC/m-NC would imply a reorganization for many IVF centers, with a greater work-load during weekends/holidays, these problems would be outweighed by the benefits on the maternal/fetal obstetric management and outcome.

Future research should be directed towards pharmacological approaches capable of compensating placental maladaptation in women who are not candidate to NC. Since common anti-platelets drugs as ASA do not seem to substantially modify this risk, the bio-molecular cascades triggered by the biomarkers object of our analyses are promising targets. Further investigation of these pathways may provide mechanistic insights and inform the development of targeted therapeutic strategies.

### Strengths and limitations

This study combines clinical and molecular analyses to provide a comprehensive assessment of obstetric outcomes and placental angiogenic profiles in OD pregnancies with different endometrial preparation strategies. Key strengths include the inclusion of both NC/m-NC and AC cycles, allowing direct comparison and the evaluation of placental gene expression to offer mechanistic insight into the role of the corpus luteum. However, some limitations should be noted. The sample size, particularly for molecular analyses, was relatively small, which may limit statistical power and generalizability of the results. Group distribution was uneven due to the predominance of AC preparation in OD cycles worldwide, potentially introducing selection bias. Finally, the observational design precludes causal inference and logistical differences across IVF centers may limit the broader applicability of NC/m-NC protocols. Despite these limitations, the findings provide valuable hypothesis-generating insights and a rationale for future research.

## Conclusions

An increase of OD pregnancies and related complications is expected in the coming years being a challenge for all obstetricians and neonatologists. Therefore, all possible strategies to improve maternal-fetal outcomes should be adopted. Herein, we report that NC/m-NC presents a lower risk of developing hypertensive complications and placental anomalies, with positive consequences on both maternal/fetal side. Angiogenic biomarkers involved in placental development could justify these findings and potentially offer a treatment perspective for those women in whom artificial preparation of the endometrium represents the only available strategy.

## Data Availability

The datasets used and/or analysed during the current study are available from the corresponding author on reasonable request.

## Abbreviations

AC: Artificial cycle
ASA: Acetylsalicylic acid
CL: Corpus luteum
CS: Cesarean section
CTRL: Control
ELISA: Enzyme-linked immunosorbent assay
FET: Frozen embryo-transfer
GDM: Gestational diabetes
HDP: Hypertensive disorders of pregnancy
ICSI: Intracytoplasmic sperm injection
IQR: Interquartile range
IUGR: Intrauterine growth restriction
IVF: In vitro fertilization
LBW: Low birth weight
LGA: Large for gestational age
m-NCs: Modified natural cycle
NC: Natural cycle
NICU: Neonatal intensive care unit
OD: Oocyte donation
PAPP-A: Pregnancy-associated plasma protein
PE: Pre-eclampsia
PIH: Pregnancy-induced hypertension
PlGF: Placental growth factor
PPH: Post-partum haemorrage
pPROM: Preterm premature rupture of membranes
PTB: Preterm birth
RIPA: Radio immunoprecipitation assay
SD: Standard deviation
sFlt-1: Soluble FMS-like tyrosine kinase-1
SGA: Small for gestational age
VEGF: Vascular endothelial growth factor
